# Parent and child mental health during COVID-19 in Australia: The role of pet attachment

**DOI:** 10.1371/journal.pone.0271687

**Published:** 2022-07-25

**Authors:** Shannon K. Bennetts, Sharinne B. Crawford, Tiffani J. Howell, Fiona Burgemeister, Catherine Chamberlain, Kylie Burke, Jan M. Nicholson

**Affiliations:** 1 Judith Lumley Centre, La Trobe University, Melbourne, Australia; 2 Intergenerational Health Group, Murdoch Children’s Research Institute, Melbourne, Australia; 3 Anthrozoology Research Group, School of Psychology and Public Health, La Trobe University, Bendigo, Victoria, Australia; 4 NGANGK YIRA: Murdoch University Research Centre for Aboriginal Health and Social Equity, Perth, Australia; 5 Metro North Mental Health, Royal Brisbane and Women’s Hospital, Herston, Queensland, Australia; University of Jyvaskyla, FINLAND

## Abstract

Restrictions, social isolation, and uncertainty related to the global COVID-19 pandemic have disrupted the ways that parents and children maintain family routines, health, and wellbeing. Companion animals (pets) can be a critical source of comfort during traumatic experiences, although changes to family routines, such as those caused by COVID-19, can also bring about challenges like managing undesirable pet behaviours or pet-human interactions. We aimed to examine the relationship between pet attachment and mental health for both parents and their children during the COVID-19 pandemic in Australia. A total of 1,034 parents living with a child under 18 years and a cat or dog completed an online cross-sectional survey between July and October 2020. Path analysis using multivariate linear regression was conducted to examine associations between objective COVID-19 impacts, subjective worry about COVID-19, human-pet attachment, and mental health. After adjusting for core demographic factors, stronger pet-child attachment was associated with greater child anxiety (parent-reported, *p* < .001). Parent-pet attachment was not associated with self-reported psychological distress (*p* = .42), however, parents who reported a strong emotional closeness with their pet reported greater psychological distress (*p* = .002). Findings highlight the role of pets during times of change and uncertainty. It is possible that families are turning to animals as a source of comfort, during a time when traditional social supports are less accessible. Alternatively, strong pet attachment is likely to reflect high levels of empathy, which might increase vulnerability to psychological distress. Longitudinal evidence is required to delineate the mechanisms underpinning pet attachment and mental health.

## Introduction

Humans are innately social beings; we have a need for regular, ongoing, and meaningful social connections and support [1]. Social isolation can contribute to feelings of loneliness, boredom, anxiety, and depression, especially for children and adolescents [2]. The COVID-19 pandemic, an ongoing “public health emergency of international concern” [3], has prompted widespread and significant changes to the ways that we work, live and study, requiring forced isolation and disrupting opportunities to engage in social connections that keep us mentally well [4]. Australian efforts to curb transmission of the SARS-CoV-2 virus which causes COVID-19 have included strict ‘stay at home’ orders, remote school learning and working, curfews, business closures, and restrictions on movement [5]. With families spending more time at home than ever before, there has been extraordinary demand for pet adoptions [6] and considerable media attention on the role of pets during times of change and uncertainty [7]. Using a large online survey of Australian parents with children and at least one cat or dog, we report here some of the first evidence about the role of pet attachment for parent and child mental wellbeing during the COVID-19 pandemic.

Attachment refers to the deep and enduring emotional connections we form–typically between two people—in which each seeks closeness and feels more secure when the attachment figure is present. Attachment theory was originally used to conceptualise infant-parent relationships [8], but is now considered in a broad range of contexts, including pet-human relationships [9].

Australia has one of the highest rates of pet ownership worldwide, with nearly two-thirds of households (61%) owning a pet in 2019, including at least 5.1 million dogs and 3.8 million cats [10]. In comparison, pet ownership rates are estimated to be around 57% in the United States and 40% in the United Kingdom [10]. The potential for companion animals (‘pets’) to enhance human wellbeing has been well-documented, including both psychological and physical benefits such as enhanced self-esteem, increased physical activity, improved hormonal levels, and reduced heart rate [11, 12].

For children, interacting with pets has the potential for myriad benefits, including better physical health, more physical activity, less parental concern about child mood, behaviour, and learning ability, and better emotion regulation [13, 14]. Pets can offer unconditional and non-judgemental companionship, which might be particularly beneficial for those with pre-existing mental health conditions or those who have experienced trauma [15, 16]. Longitudinal evidence has also demonstrated that having a family pet can protect children from developing socio-emotional problems, especially for children without siblings [17].

While the benefits of pets for wellbeing are numerous, it is important to acknowledge the mixed research evidence. A systematic review of 17 studies examining the association between companion animals and mental health found positive, negative and neutral impacts of pet ownership [15]. The review highlighted challenges such as the practical and emotional burdens of pet ownership, as well as the emotional impact of the pet passing away. Cats and dogs can also disrupt sleep [18], exhibit challenging behaviours such as aggression [19], and they are sensitive to changes in their routines and home environment [20]. Strong pet attachment has been associated with more symptoms of psychological distress, including depression and anxiety [21]. Similarly, stronger pet attachment has been linked to poorer mental health for elderly populations [22] and workers in high-risk occupations such as emergency services [23].

In addition to the health impacts of contracting COVID-19, restrictions across Australia, as elsewhere in the world, have caused significant and widespread economic and social upheaval [24]. A representative survey in March 2020 found that Australian adults were experiencing substantially elevated anxiety and depression symptoms, compared to population norms, and that financial distress was strongly related to poorer mental health [25]. Parents are experiencing greater stress and poorer mental health during the pandemic, but greater perceived parental control and parental support can be protective [26]. Social isolation, loss of income, and less access to support can make it harder to look after physical and mental health and wellbeing [27]. Many parents are working from home, while simultaneously supporting their children to study from home. As a group, Australian parents have reported lower subjective wellbeing, compared to pre-pandemic, especially for families with pre-existing mental health risks, socioeconomic disadvantage, or pandemic-related work impacts [28].

So, how does human-pet attachment relate to parent and child mental wellbeing during a global pandemic? Evidence is beginning to emerge. For example, open-ended responses from a 2020 survey revealed that US dog owners felt that dogs had contributed to reducing feelings of loneliness and isolation and protected their mental and physical health [29]. Conversely, quantitative data from US pet owners suggested that the impact of pet attachment differed by pre-pandemic mental health status; pet attachment was protective for those with moderate or high distress, but those with strong pet attachment and severe distress were likely to continue to experience severe distress [30]. Evidence from the UK lockdown has shown that greater pet attachment was associated with more symptoms of psychological distress, including depression and anxiety [31]. The contribution of pets for mental health is an increasingly salient issue given the global surge in demand for pet adoptions, evidenced by a 250% increase in Google searches for cat and dog adoptions between 2019 and 2020 [32].

The COVID-19 pandemic presents a unique opportunity to understand how families manage during a time of rapid societal change and uncertainty. The overarching aim of this study was to examine associations between human-pet attachment and mental health, for both parents and children, in the context of a global pandemic. In doing so, we firstly examine objective COVID-19 impact and subjective COVID-19 worry and consider how these flow on to associations with pet attachment and mental health. Given the mixed evidence regarding the role of pets for mental health, we propose five research questions, rather than specific hypotheses. These research questions correspond to five pathways in a model that we introduce in the method, tested in relation to both parents and children.

Path 1: What is the association between objective COVID-19 impact and subjective COVID-19 worry?

Path 2: What is the association between objective COVID-19 impact and mental health?

Path 3: What is the association between subjective COVID-19 worry and pet attachment?

Path 4: What is the association between subjective COVID-19 worry and mental health?

Path 5: What is the association between pet attachment and mental health?

Findings will help to improve our understanding of how companion animals may enhance, or detract from, parent/child mental wellbeing.

## Materials and methods

### Design

Data for the Parents, Pets & Pandemic Survey were collected over a 12-week period, from July 2020 to October 2020, using an online cross-sectional design. Eligibility criteria required that participants were: (i) over 18 years old; (i) living in Australia; (iii) living with at least one child under 18 years at least some of the time; and (iii) living with at least one cat or dog. There were no additional exclusion criteria; participants were only required to meet the four inclusion criteria. Ethical approval was granted by the La Trobe Human Research Ethics Committee (HEC20251). Participants were asked to tick a checkbox to indicate their electronic consent, after being provided with an electronic copy of the Participant Information Statement.

The survey was hosted by REDCap, a secure web-based data capture platform [33] and we also created a study Facebook page for recruitment purposes. Reporting on this survey was guided by the Checklist for Reporting Results of Internet E-Surveys (CHERRIES) [34]. Deidentified data and analytic code will be made available upon request, subject to ethical approval.

### Context

Data collection coincided with the ‘second wave’ of COVID-19 in Australia, which most notably impacted the state of Victoria. By late October 2020 (the end of our data collection period) Australia had recorded 27,590 cases and 907 deaths. The largest number of national daily cases during the survey was 698 (5^th^ August) and the largest number of daily deaths was 59 (4^th^ September). Although these figures were low compared to most countries at the time, strict measures were implemented to minimise transmission. The 4.9 million residents of Melbourne, the capital of Victoria, were subject to a heavy lockdown between July and October 2020. During this time, there were only four permitted reasons for leaving home: (i) essential work; (ii) purchasing essential supplies or services; (iii) restricted physical exercise for no more than one hour per day; (iv) receiving or providing care. For part of this time, residents were also subject to a night-time curfew and could not move more than 5km beyond their place of residence. The remaining Australian states and territories were experiencing very low numbers of COVID-19 infections and were subject to some restrictions (e.g., masks, social distancing, limits on public and private gatherings).

### Measures

A team of researchers with expertise in parenting, mental health, public health, and animal-human interaction selected the measures and tested the survey. The survey included 107 closed-ended items and 2 open-ended items and took approximately 15 minutes to complete. All survey items were optional, except for several mandatory demographic items (e.g., gender, state). To avoid selection bias and minimise participant burden, participants with more than one child under 18 years were asked to respond about the child with the next birthday (“focus child”). Participants with more than one cat or dog were asked to respond about the cat or dog who most recently joined the family (“focus pet”). Although we acknowledge that human-pet attachments are likely to vary for different pets, this decision was made to avoid bias in pet selection, to minimise the burden of multi-pet responding, and to capture new pets who had been acquired during the pandemic. Participants were asked to enter the first name, initial, or a nickname for both their child and their pet. This information was auto-populated for all child- and pet-related items, to ensure consistent reporting about the same ‘focus child’ and ‘focus pet’.

### Demographics

Parent, child, pet, and family demographics included parent/child gender, parent age (years), child age group, parent education, single parent status, only child status, non-English language spoken at home, number of adults and number of children living in the household, and Indigeneity. Participants were asked to indicate their location (one of eight Australian states and territories). Based on participant postcodes, we classified participants’ location as metropolitan or non-metropolitan, as well as generating a neighbourhood disadvantage variable using the Index of Relative Socioeconomic Disadvantage (IRSD). The IRSD has an Australian mean of 1000 and standard deviation of 50, with lower scores indicating greater neighbourhood disadvantage [35].

### Parent and child wellbeing

Parent and child psychological wellbeing were assessed using brief, validated measures. The K6 [36] is a 6-item measure of psychological distress for adults, administered on a 5-point scale, where higher scores indicate greater distress (e.g., “nervous”). Australian K6 scoring produces a possible score between 6 and 30, with a score between 19–30 indicative of a ‘probable serious mental illness’ [37]. Child anxiety was measured using four items from the Spence Child Anxiety measure (e.g., “worries that something bad will happen to them”). Permission was obtained from Professor Susan Spence for use of these four items in the current survey.

### Human-pet attachment

A brief, validated measure of human-pet attachment was used for parents’ self-report and parent-report about their child [38]. This scale captures the frequency of human-animal interactions. Both versions comprised six items on a 5-point scale, with higher scores indicating stronger pet attachment (e.g., “How often do you spend time each day playing with or exercising your pet?”, normative data: mean and std dev: 2.9 (0.63) for children and 2.8 (7.8) for caregivers). Permission was obtained for use of these items in the current survey.

For parents only, we also administered the 12-item emotional closeness subscale of the Cat/Dog Owner Relationship Scale [C/DORS; 39] (e.g. “*[Pet]* gives me a reason to get up in the morning”). In contrast to the above pet attachment measure, this subscale captures the strength of the perceived *emotional bond* between human (adults only) and pet. We included this measure to investigate whether associations with parent mental health varied for a general/behavioural measure of the human-pet bond, compared to a measure that specifically captures emotional aspects of the human-pet bond. Separate scores are produced for cat and dog owners, which we converted to a standardised score (*z-score*) to produce a single variable for analyses.

### COVID-19 measures

A COVID-19 Impact Score was derived by summing six objective variables: diagnosed, tested, identified as a close contact, subject to mandatory isolation, living in metropolitan Melbourne (1 = yes; 0 = no), and income stress during the pandemic (1 = finding it difficult or very difficult on current income; 0 = coping or living comfortably on current income). Metropolitan Melbourne location was used as a proxy measure of impact, considering the significant restrictions in place for metropolitan Melbourne residents at the time of data collection. This resulted in a total possible Impact Score between 0–6, with higher scores indicating greater impact. A COVID-19 Worry Score was derived by summing two subjective items: one about concern for own safety and one about concern for the safety of family or close friends (each measured on a 5-point scale: 0 = not worried; 1 = a little worried; 2 = somewhat worried; 3 = quite worried; 4 = very worried). This resulted in a total possible Worry Score between 0–8, with higher scores indicating greater worry.

### Recruitment

Participants were recruited using both paid and unpaid Facebook advertising. Unpaid advertising involved posting about the survey on Facebook Pages or Groups (e.g., pet groups, parent groups), while paid advertising involved Facebook campaigns conducted via the Facebook Ads Manager. We employed an active and flexible approach to recruitment, monitoring for ‘gaps’ in participant sub-groups (e.g., fathers), and adjusting recruitment strategies accordingly. This approach is effective and necessary for social media-based research recruitment [40].

At the end of the survey, participants could choose to enter a prize draw to receive one of ten AUD$20 gift cards. The final survey page included a list of support services, which we considered to be critical, given that participation could be anonymous (and there was no direct contact with researchers) at a time of increased mental health vulnerability [41].

### Statistical analyses

**Fig 1:**
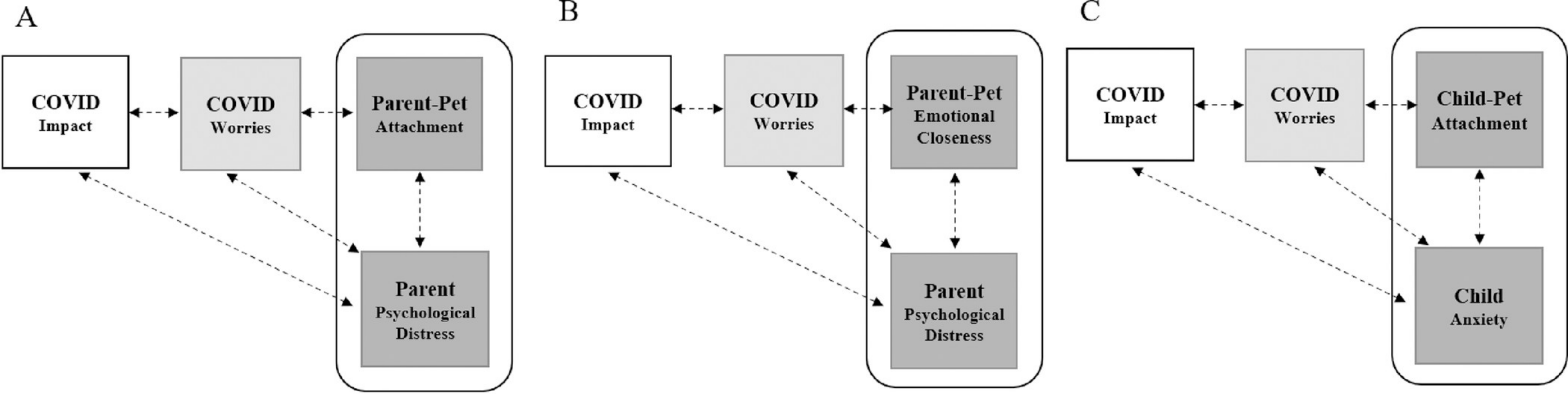
Models to be tested: (A) Parent-pet attachment, (B) Parent-pet emotional closeness, (C) Child-pet attachment.

Path analysis with linear regression was applied to test three theoretical models regarding the associations between pet attachment mental health during COVID-19 (see **Fig 1**). As outlined in the introduction, each model included five pathways of association, corresponding to the five research questions: (1) Impact and Worries, (2) Impact and Mental Health, (3) Worry and Attachment, (4) Worry and Mental Health, and (5) Attachment and Mental Health. For each model, we wanted to firstly determine whether there was a relationship between objective COVID-19 impact and parents’ subjective worry about the COVID-19 situation. We then examined whether this worry was associated with mental health and/or pet attachment, as well as the relationship between mental health and pet attachment.

Our adjusted models included 12 predictors, therefore requiring at least 146 participants, according to Tabachnick and Fidell [42] (N ≥ 50 + 8m, where *m* is the number of independent variables and N is the sample size). As shown in **Fig 1**, we tested three versions of this model: (A) parent-pet attachment and mental health (K6), parent-pet emotional closeness and mental health (K6), and child-pet attachment and mental health (Spence child anxiety scale).

We checked for evidence of multicollinearity using the *vif* command in *Stata* and checked histograms of the mental health and pet attachment variables for evidence of non-normality. Adjusted models controlled for 10 variables known to be associated with parent/child wellbeing or pet attachment: parent gender, child gender, only child status, parent age, child age group, parent education, single parent, non-English speaking background, pet type, and neighbourhood disadvantage. Given the strong association between parent and child mental health [43, 44], **Models A and B** (both parent) were adjusted for child anxiety, and **Model C** (child) was adjusted for parent psychological distress.

## Results

### Participants

**Fig 2:**
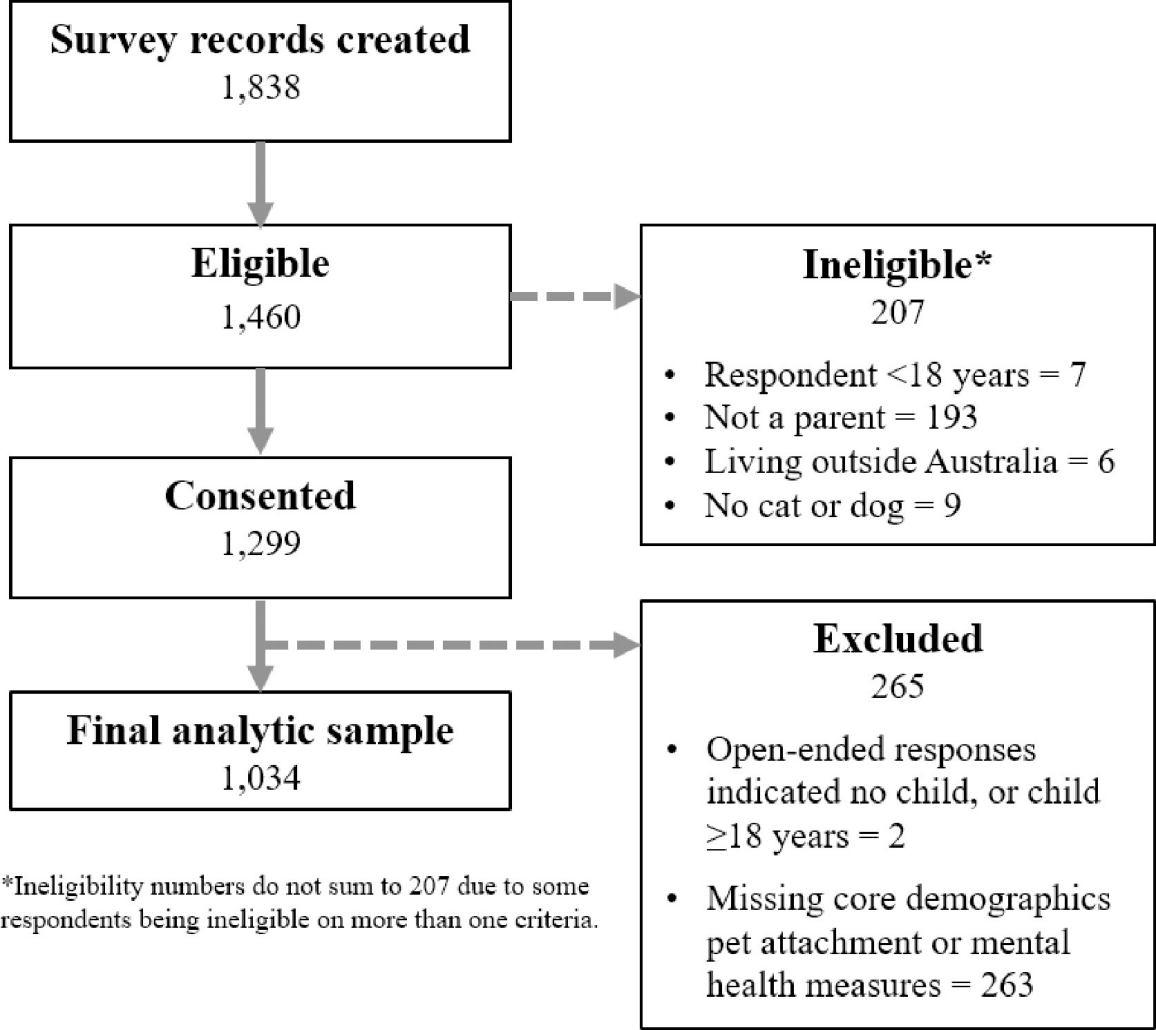
Participant flow from activation of survey link to survey completion.

A total of *N* = 1,299 participants consented to participate in the survey and the final analytic sample was 1,034 (See **Fig 2**). Facebook recruitment metrics are presented in **S1 File.** The paid Facebook campaigns reached a total of 210,520 users, generated 2,269 clicks, 659 consents and 497 completed surveys. Mean costs were 75c per click, $2.50 per consent, and $3.31 per completed survey (in AUD). As shown, 215 respondents were screened-out during the eligibility items; 90% of these (*n* = 193) were due to not having a human child under 18 years of age. Comments suggested that many of these ineligible respondents were self-identifying as parents to their ‘fur children’.

During data cleaning, 263 cases were excluded from analysis due to missing core demographic variables (i.e., parent gender, state of residence, pet type) or missing more than one primary outcome or exposure measure (i.e., parent psychological distress, child anxiety, parent-pet attachment, child-pet attachment). For the abovementioned outcome and exposure variables, cases with one item missing were replaced with the mean response option of the available items, enabling the generation of a total score (these included: K6: 24 cases; child anxiety: 10 cases; parent-pet attachment: 15 cases; child-pet attachment: 32 cases; parent-pet emotional closeness: 32 cases). Two additional cases were excluded because free-text responses indicated that the respondents did not have a child under 18 years of age.

Internal consistency of key scales was acceptable to good: child anxiety (4 items): *α* = .80; parent psychological distress (6 items): *α* = .88; parent-pet attachment (6 items): *α* = .71; child-pet attachment (6 items): *α* = .83; parent emotional closeness with pet: *α* = .85 (cat, 11 items), *α* = .88 (dog, 10 items).

Demographics for the analytic sample are presented in **Table 1**. Most participants were female (78%); however, the targeted Facebook advertising was effective for boosting male participant numbers, who were initially difficult to engage/poorly represented. Most participants had a tertiary qualification. Only a small proportion identified as Indigenous (1%) or spoke a non-English language at home (7%). Focus children (those with the next birthday) represented a range of ages, with the largest group aged 10–14 years (38%), roughly half female and male, and one-third were the family’s only child. For nearly two-thirds of respondents, the focus pet was a dog (most recently acquired cat or dog). Two-thirds of families were living in Victoria and the remaining third were based in other Australian states and territories. Half were living in a metropolitan location in Australia, and one-quarter were living in metropolitan Melbourne. Most respondents were living in two-parent families (81%) and in less disadvantaged neighbourhoods than the Australian mean of 1000. One-fifth had introduced a new cat or dog during the COVID-19 pandemic and a small number reported that a pet had died or been surrendered. Reasons for introducing a new pet during this time (since March 2020) included: for children’s health and wellbeing (35.9%), extra time to help the new pet settle in (26.70%), for the parents’ health and wellbeing (25.7%), children were asking for a pet (25.2%), and to help children learn responsibility (19.9%). Descriptives for the COVID-19, pet attachment, and mental wellbeing measures are presented in **Table 2**. Overall, the majority of respondents had been tested for COVID-19 but very few had contracted the virus.

**Table 1:** Parent, child, pet and family characteristics (*n* = 1,034).

Parent	*n (%)*	*Mean (SD)*	*Range*
Parent age, years	-	43.0 (6.9)	20–65
Parent gender			
Female	803 (77.7)		
Male	225 (21.8)		
Non-binary	1 (0.1)		
Prefer not to say	5 (0.5)		
Without tertiary education	387 (38.4)		
Single parent	164 (15.9)		
Indigenous	14 (1.4)		
LOTE^	74 (7.2)		
**Focus Child**			
Child age, years			
0–4	144 (13.9)		
5–9	277 (26.8)		
10–14	395 (38.2)		
15–17	218 (21.1)		
Child gender			
Female	489 (47.3)		
Male	537 (51.9)		
Non-binary	5 (0.5)		
Prefer not to say	3 (0.3)		
Only child	355 (34.5)		
**Pet**			
Focus pet type			
Cat	364 (35.2)		
Dog	670 (64.8)		
During COVID-19 (since March)			
Got a new pet	206 (20.0)		
Pet died	82 (8.0)		
Pet surrendered	6 (0.6)		
**Family**			
Location			
Victoria	636 (61.5)		
Outside Victoria	398 (38.5)		
Metropolitan Melbourne	267 (25.8)		
Neighbourhood Disadvantage*	-	1022 (59)	838–1128

^Language Other Than English spoken at home.

*Index of Relative Socioeconomic Disadvantage (IRSD) which has an Australian mean of 1000 and standard deviation of 50, with lower scores indicating greater relative disadvantage. n = number, sd = standard deviation.

**Table 2:** Sample descriptives for COVID-19 impact, worry, parent and child mental health, and parent and child attachment to pet (*n* = 1,034).

	Possible Range	Total	Vic Metro N = 267	Other Location N = 767
**COVID-19 Impact**				
Diagnosed, n (%)	n/a	3 (0.3)	2 (0.8)	1 (0.1)
Tested, n (%)	n/a	624 (60.5)	176 (66.2)	448 (58.6)
Close contact, n (%)	n/a	29 (2.8)	14 (5.2)	15 (2.0)
Isolated, n (%)	n/a	371 (36.0)	95 (35.7)	276 (36.0)
High income stress, n (%)	n/a	114 (11.1)	22 (8.3)	92 (12.1)
Total Impact Score, m (sd)	0–4	1.4 (1.0)	2.2 (0.9)	1.1 (0.9)
**COVID-19 Worry**				
Worry about self, m (sd)	0–4	1.9 (1.1)	1.9 (1.1)	1.9 (1.1)
Worry about others (high)	0–4	2.6 (1.1)	2.6 (1.1)	2.6 (1.1)
Total Worry Score, m (sd)	0–8	2.2 (2.1)	4.4 (2.0)	2.2 (2.1)
**Parent psychological distress**				
Total score, m (sd)	6–30	12.8 (5.1)	13.0 (4.5)	12.7 (5.3)
Within clinical range, n (%)	n/a	140 (13.6)	31 (11.6)	109 (14.3)
**Child anxiety**	4–16	6.8 (2.4)	6.6 (2.1)	6.9 (2.5)
**Parent-pet attachment**	1–4	3.3 (0.5)	3.2 (0.5)	3.3 (0.5)
**Parent-pet emotional closeness, m (sd)^**				
Cat	1–5	3.4 (0.7)	3.5 (0.7)	3.4 (0.8)
Dog	1–5	3.7 (0.7)	3.6 (0.7)	3.8 (0.7)
**Child-pet attachment**	1–4	3.1 (0.6)	2.9 (0.6)	3.1 (0.6)

^Z-Score used in models, n/a = not applicable, n = number, m = mean, sd = standard deviation.

### Correlations between COVID-19 impact and worry, pet attachment and mental wellbeing

Correlations between COVID-19 impact and worry, pet attachment, and mental wellbeing are presented in **Table 3**. There was no association between parent-pet attachment and parent psychological distress. There was a small but significant association between objective COVID-19 impact and worry. COVID-19 worry was positively associated with all pet attachment and mental health variables, although coefficients were small. The strongest correlation was between parent-pet attachment and parent emotional closeness with pet, which is not surprising given that these are similar constructs. There was also a moderately strong association between parent-pet and child-pet attachment. There was no association between parents’ psychological distress and parent-pet attachment, but parent-pet emotional closeness was associated with greater psychological distress. Lastly, stronger (parent-reported) child-pet attachment was associated with greater child anxiety.

**Table 3:** Correlations between COVID-19 impact, worries, parent and child mental health, and parent and child attachment to pet (*n* = 1,034).

	1.	2.	3.	4.	5.	6.
**1**. COVID-19 Impact	-					
**2**. COVID-19 Worry	**.09** **	-				
**3**. Parent Psychological Distress	**.13** ***	**.23** ***	**-**			
**4**. Child Anxiety	.06	**.22** ***	**.33** ***	**-**		
**5**. Parent-Pet Attachment	-.06	**.12** ***	-.02	.05	**-**	
**6**. Parent-Pet Emotional Closeness	-.03	**.18** ***	**.14** ***	**.09** **	**.62** ***	**-**
**7**. Child-Pet Attachment	-.04	**.16** ***	.02	**.21** ***	**.47** ***	**.35** ***

*p < .05

**p < .01

***p < .001.

Note: Correlation coefficients reported here are the more conservative Spearman’s, due to some skewness affecting the K6, child anxiety, parent-pet attachment, child-pet attachment. Multicollinearity was checked using *vif* command; no evidence of multicollinearity was observed.

### Path models testing associations between pet attachment and mental wellbeing

**Fig 3:**
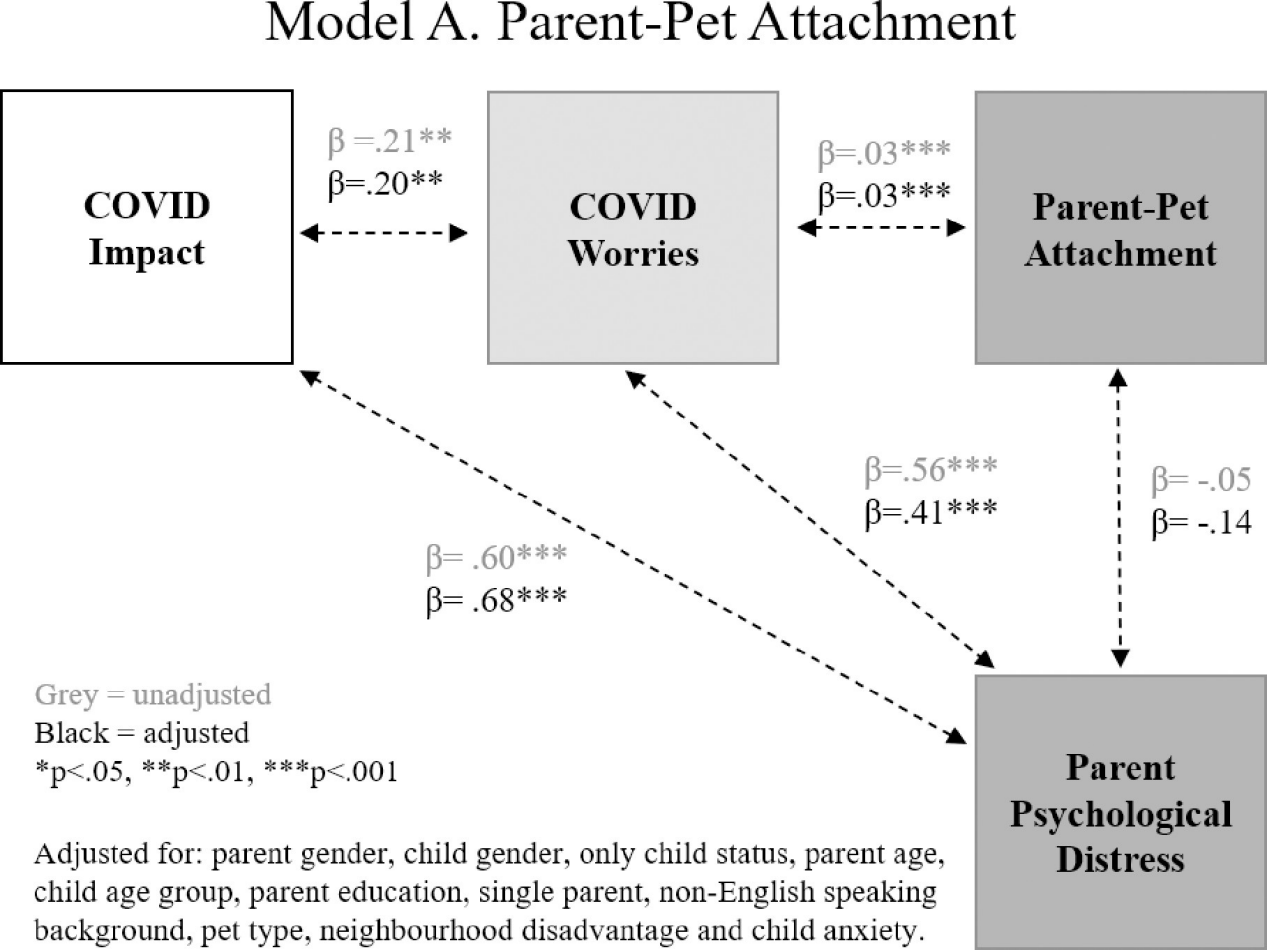
Model A: Associations between COVID-19 impact and worries, parent-pet attachment and parent mental health.

**Fig 4:**
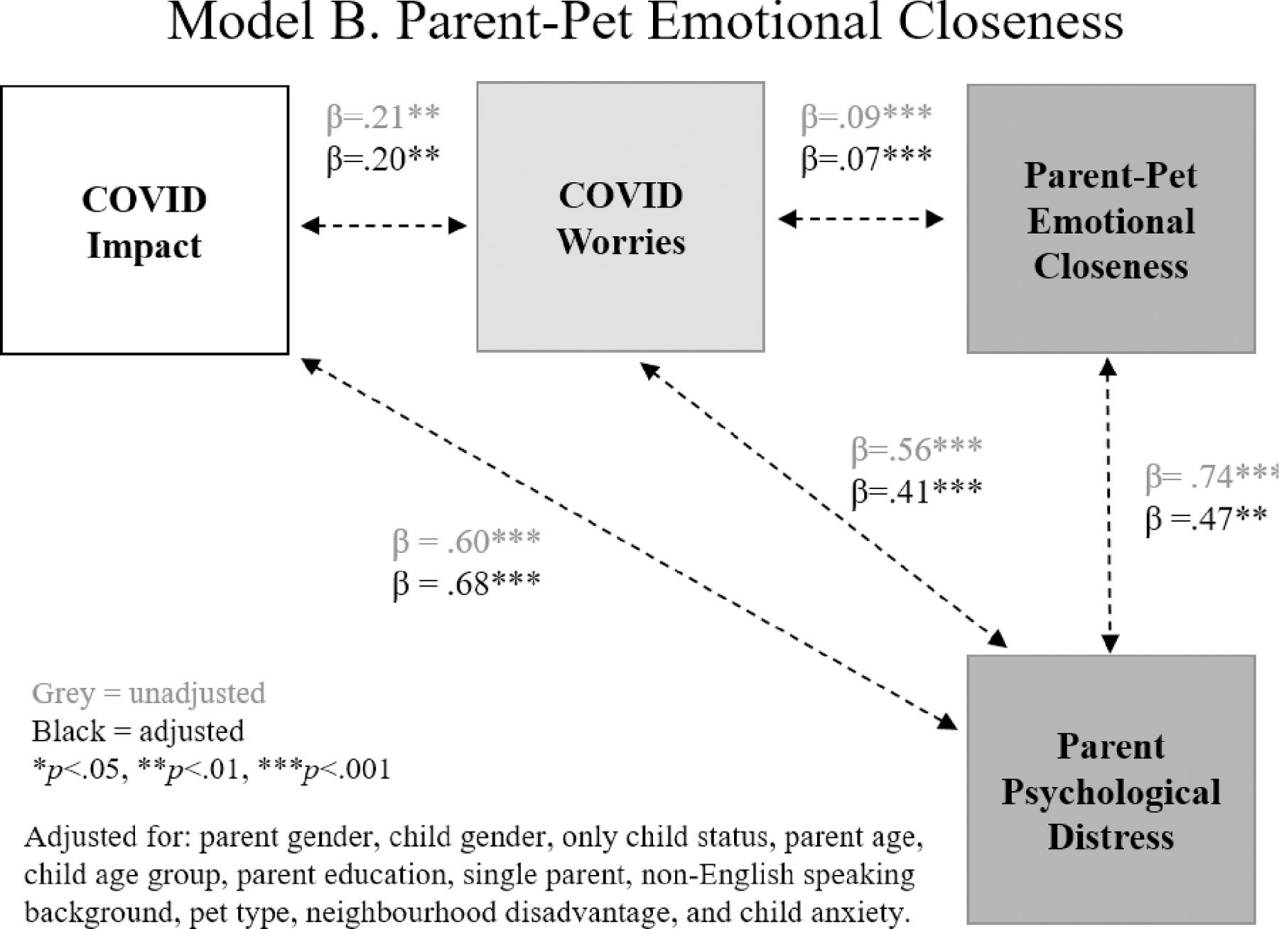
Model B: Associations between COVID-19 impact and worries, parent-pet emotional closeness and parent mental health.

**Fig 5:**
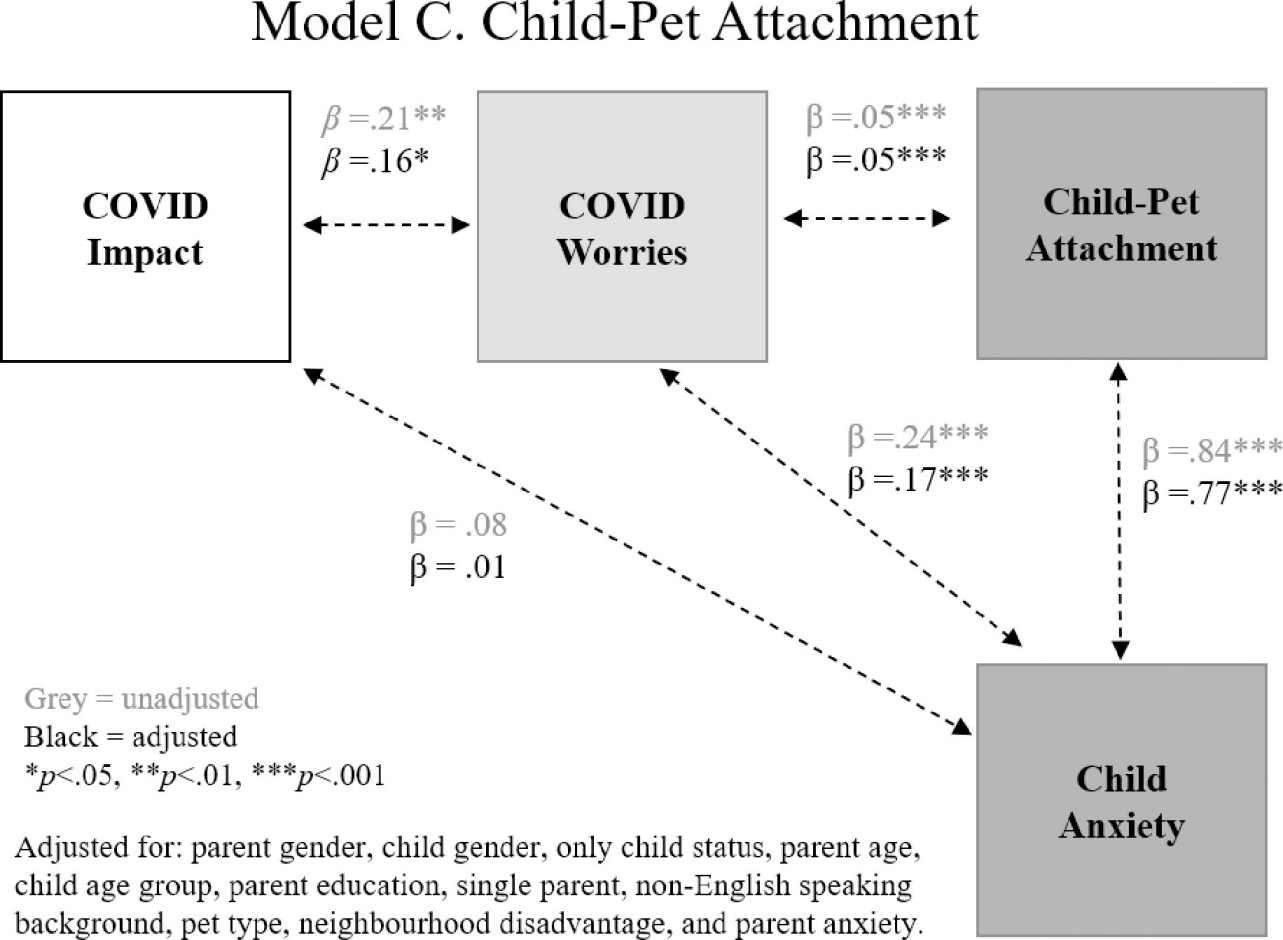
Model C: Associations between COVID-19 impact and worries, child-pet attachment and child anxiety.

Results of the three path models are shown in **Figs 3–5**, related to: (i) parent-pet attachment, (ii) parent-pet emotional closeness, and (iii) child-pet attachment. In each model, greater objective COVID-19 impacts were associated with greater parent worry about COVID-19. Greater impact was also associated with greater parent psychological distress but was not related to child anxiety. As shown in **Fig 3**, greater worry was associated with stronger parent-pet attachment and greater psychological distress, however there was no association between parent-pet attachment and mental health. As shown in **Fig 4**, greater COVID-19 worry was linked to stronger emotional closeness with pet and poorer mental health. Unlike parent-pet attachment, greater emotional closeness with pet was associated with greater psychological distress. Finally, **Fig 5** shows that greater child-pet attachment was linked to greater child anxiety.

## Discussion

We conducted a large Australia-wide survey of parents with a cat or dog during the ‘second wave’ of the COVID-19 pandemic in 2020, to understand the relationship between pet attachment and parent/child mental health, as well as associations with pandemic impacts and worries. We addressed three research questions related to mental health and parent-pet attachment, parent-pet emotional closeness, and child-pet attachment. Pet attachment in the current sample was slightly stronger than the US measure development sample [38]. As would be expected, greater COVID-19 impact (e.g., tested for COVID-19, experiencing income stress) was associated with greater COVID-19 worry (for self and others), although the association was small and perhaps speaks to individual circumstances and perceptions of risk.

Path modelling indicated that the effects of COVID-19 were exerting influence on mental health directly, and via pet attachment. We found that more anxious children (parent-reported) had stronger pet attachment, and more distressed parents had a greater emotional closeness with their pet. These associations attenuated slightly after controlling for parent/child mental health, reflecting environmental and genetic origins of anxiety, whereby children are more likely to experience anxiety if their parents do [45]. Although this finding might seem counter-intuitive, due to evidence that pets are health-promoting [e.g., 17, 46], this negative association between pet attachment and mental health has been reported elsewhere, both generally and during COVID-19 [e.g., 21–23, 31]. Given that causality cannot be inferred, there are two possibilities. First, parents and children who were feeling unsettled during the pandemic were gravitating towards pets to seek additional comfort. Second, stronger pet attachment is contributing to greater distress. The reality may reflect a combination of the two. The pandemic has created a highly stressful environment for some families who are negotiating the challenges of working and learning from home with pets, operating in the absence of their usual social supports and outlets. This environment is particularly difficult for those with pre-existing physical or mental health difficulties. Our findings concur with work by Ratschen and colleagues [31], who concluded that strong human-animal bonds might be linked to mental health vulnerabilities. It is possible that strong pet attachment reflects a capacity for high levels of empathy, particularly towards animals. Findings might also speak to personality traits or coping skills of parents or children, such that pets are viewed as offering a “safe haven” or “secure base” [47].

Interestingly, there was no relationship between parent-pet attachment and parents’ mental health. This might be due to the nature of the survey items; the 6-item pet attachment measure has a more behavioural focus (e.g., “How often do you have your pet near you when you study, read, or watch TV?”) whereas the emotional closeness measure is more closely aligned with aspects of mental health and empathy (e.g., “How traumatic do you think it will be for your when your pet dies?”). It is therefore likely that the inconsistent body of evidence related to pet attachment and mental health is, to a large extent, dependent upon the definition of attachment and/or the measures used.

One in five respondents had acquired a new cat or dog since the pandemic commenced in March 2020; of these, many attributed the timing of the decision to family wellbeing. This suggests that some families were specifically seeking pets to aid coping and wellbeing. Of note, there were 193 parents (10.5% of activated survey records) who screened-out of the survey due to not having a human child under 18 years. This ostensibly speaks to the many pet owners who viewed the advertisements and self-identified as a ‘parent’, despite not having a human child. Indeed, we received several comments on Facebook advertisements to support this assertion: e.g. “*My dog is my child*” and “*I have triplets; they’re all poodles*”. Relatedly, those responding to a survey about pets may be more likely to identify as ‘animal lovers’, with strong pet attachment and enduring emotional bonds. As a result, the current sample may overestimate the strength of human-pet attachments, compared to the Australian population of cat and dog owners.

Strengths of this study are the use of a relatively large and reasonably representative sample of Australian parents with children and a cat or dog, well-validated measures of pet attachment and mental wellbeing, as well as the use of both objective and subjective COVID-19 measures. The C/DORS arose from the Monash Dog-Owner Relationship Scale, and the Cat-Owner Relationship Scale. While these individual scales had been previously validated, the C/DORS itself had not been validated at the time of data collection for this study so further work is required in this space. We extend previous evidence regarding family pets by capturing the strength of human-pet bonds (i.e., attachment) rather than just the presence or absence of a pet (i.e., ownership) [17]. The sample showed good representativeness on most demographic characteristics. For example, our sample included 18% single parents, compared to 14% in population data provided by the Australian Bureau of Statistics [48]. Our proportion of 35% cat and 65% dog owners also compares well to a recently-conducted Australian survey of pet owners [10]. Overall, half the sample was recruited via paid Facebook advertising, and half via unpaid Facebook advertising (e.g., cross-posting). Paid recruitment was effective for boosting sub-samples that were initially under-represented, such as fathers and parents in non-Victorian states.

We acknowledge several limitations. To minimise participant burden and reporting bias, respondents were asked to report on one child (i.e., child with the next birthday) and one pet (i.e., the one most recently acquired). However, these processes do not operate in isolation; pet attachment and mental health outcomes are likely to vary according to the child and pet selected, and we were unable to account for dynamics between parents, children, and pets. For example, parents and children might have the strongest attachment to an older pet, rather than the most recently acquired pet. The survey was also restricted to families with a cat or dog, due to the availability of species-appropriate attachment measures. As such, the role of other pets during the pandemic, such as chickens or rabbits, requires further investigation. Lastly, it is possible that children’s perception of pet attachment and their own anxiety might differ from parents’ perceptions. Further research should consider children’s voices.

Our findings contribute to a small body of emerging evidence regarding the role of pets for families with children during the COVID-19 pandemic. We demonstrate that greater objective COVID-19 impacts are associated with greater worry, and this worry feeds through to pet attachment and mental health outcomes. In this sample, stronger child-pet attachment and parent emotional closeness with pet were linked to poorer mental health. Longitudinal and qualitative evidence is required to extrapolate the mechanisms underlying pet attachment and family mental health, especially given the ongoing and significant social, psychological, and biological impacts of COVID-19. Findings have important implications for public health approaches, policy, and clinical support. While pet owners commonly report that their pets provide valuable psychological and social support, a very strong human-pet bond might be an indicator of mental health vulnerability and/or a lack of sufficient human social supports. Policy and public health approaches to disaster management should consider the critical role of pets for parents and children in distress, especially given the significant economic costs of poor mental health which can lead to low employment and education and a loss of earnings over the lifecourse [49]. It may be helpful for clinicians to draw on human-pet bonds as an opportunity for engagement, and to explore supplementary opportunities for self-care and human connection.

## Supporting information

S1 File Paid Facebook advertising metrics. (DOCX)
