# Supplementary material for: Parent and child mental health during COVID-19 in Australia: The role of pet attachment
Source: PLoS One. 2022 Jul 25;17(7):e0271687. doi: 10.1371/journal.pone.0271687 (PMC9312405; doi:10.1371/journal.pone.0271687)
Supplement: S1 File — (DOCX) [file pone.0271687.s001.docx]

| **Campaign** | **Dates** | **Times** | **Reach** | **Impressions** | **Freq.** | **Clicks** | **Reach-to-Click** | **Consents** | **Click-to-Consent** | **Complete** | **Costs** | | | |
| --- | --- | --- | --- | --- | --- | --- | --- | --- | --- | --- | --- | --- | --- | --- |
|  |  |  |  |  |  |  |  |  |  |  | *TOTAL* | *Per Click* | *Per Consent* | *Per Complete* |
| **C1, Dads** | 4-6 Sep | 12-2, 6-10 | 6,464 | 7,928 | 1.23 | 49 | 0.76% | 3 | 6.12% | 3 | $50.00 | 92c | $16.67 | $16.67 |
| **C2, Dads** | 7-11 Sep | 12-2, 6-10 | 6,272 | 10,176 | 1.35 | 58 | 0.92% | 4 | 6.90% | 2 | $50.00 | 78c | $12.50 | $25.00 |
| **C3, Non-Vic** | 12-18 Sep | 12-2, 6-10 | 8,059 | 19,851 | 1.31 | 139 | 1.72% | 14 | 10.07% | 7 | $100.00 | 65c | $7.14 | $14.29 |
| **C4, Dads** | 24-28 Sep | 12-2, 6-10 | 27,056 | 32,818 | 1.31 | 234 | 0.86% | 24 | 10.26% | 18 | $207.63 | 66c | $8.65 | $11.54 |
| **C5, Non-Vic** | 28-29 Sep | 12-2, 6-10 | 8,462 | 40,461 | 1.12 | 53 | 0.63% | 6 | 11.32% | 4 | $50.00 | 94c | $8.33 | $12.50 |
| **C6, Dads** | 1-3 Oct | 5-midnight | 31,905 | 40,461 | 1.27 | 246 | 0.77% | 50 | 20.33% | 37 | $198 | 73c | $3.96 | $5.35 |
| **C7, Non-Vic** | 1-3 Oct | 5-midnight | 27,854 | 33,629 | 1.21 | 255 | 0.92% | 50 | 19.61% | 34 | $198 | 71c | $3.98 | $5.82 |
| **C8, Dads** | 7-10 Oct | 5-midnight | 51,984 | 68,735 | 1.32 | 537 | 0.78% | 149 | 27.75% | 114 | $396 | 67c | $2.66 | $3.47 |
| **C9, Non-Vic** | 7-10 Oct | 5-midnight | 42,464 | 51,361 | 1.21 | 698 | 1.14% | 359 | 51.43% | 278 | $396 | 52c | $1.10 | $1.42 |
| **C10, Dads** | 15-16 Oct | 7-midnight | 11,132 | 12,663 | 1.14 | 80 | 0.72% | 15 | 18.75% | 13 | $95 | 1.20 | $6.33 | $7.31 |
| **C11, Non-Vic** | 15-16 Oct | 7-midnight | 9,520 | 9,953 | 1.05 | 106 | 1.11% | 29 | 27.36% | 21 | $95 | 90c | $3.28 | $4.52 |
| **C12, Dads** | 26-28 Oct | anytime | 25,412 | 26,787 | 1.04 | 241 | 0.95% | 9 | 3.73% | 9 | $202.61 | 77c | $22.50 | $22.50 |
| **C13, Non-Vic** | 26-28 Oct | anytime | 20,202 | 21,108 | 1.04 | 174 | 0.86% | 23 | 13.22% | 18 | $154.68 | 89c | $6.73 | $8.59 |
| **TOTAL** | - | - | 210,520 | 305,420 | 1.26 | 2,269 | 1.08% | 659 | 21.04% | 497 | $2,192.91 | 75c | $2.50 | $3.31 |

**Supplementary File 1.** Paid Facebook Advertising Metrics.

Note: All costs are in AUD. The same images were used for campaigns 6, 8, 10, 12 and for campaigns 7, 9, 11, 13. New images were briefly tested for campaigns 12 and 13. Total cost includes tax.
